# Spintronic Transport in Armchair Graphene Nanoribbon with Ferromagnetic Electrodes: Half-Metallic Properties

**DOI:** 10.1186/s11671-016-1673-5

**Published:** 2016-10-13

**Authors:** Hongmei Liu, Hisashi Kondo, Takahisa Ohno

**Affiliations:** 1 Institute of Condensed Matter Physics, Linyi University, Shuangling Road, Linyi, 276000 Shandong People’s Republic of China; 2 CMSU, National Institute for Materials Science, Tsukuba, Ibaraki 305-0047 Japan; 3 Institute of Industrial Science, University of Tokyo, Meguro, Tokyo, 153-8505 Japan

**Keywords:** Graphene nanoribbon, Graphene-nickel contact, Spin filter, Electron transport

## Abstract

Utilizing first-principles theory, we demonstrate that half-metallicity can be realized in a junction composed of non-magnetic armchair graphene nanoribbon (AGNR) and ferromagnetic Ni electrodes. The half-metallic property originates from the AGNR energy gap of the up spin located at the Fermi energy, while large electronic states are generated for the down spin. By altering the interlayer distance and the contact area, namely, the strength of AGNR-Ni interaction, the efficiency of the spin filter becomes lower, since the energy gap moves away from the Fermi energy with the variation of charge transfer intensity.

## Background

Spintronic nanodevices, which are nanoscale devices utilizing spin degrees of freedom of electrons, have attracted a significant amount of attention. One of the significant functions of spintronics is the spin filter effect. High spin filter efficiency is expected to be realized in half-metallic materials with one metallic spin component and the other semiconducting or insulating spin channel. Up to now, half-metallic property has been found not only in some ferromagnetic metals, such as manganese perovskites [1] and Heusler compounds [2], but also in some metal-free materials, for example, carbon nanomaterials [3, 4] and graphitic carbon nitride [5]. Exploring half-metallic materials is of great interest in future spintronic devices, but it still remains a challenge due to the requirement of unique spin-asymmetric electronic states.

Carbon-based nanomaterials, such as graphene, are expected to be promising materials for the realization of spintronics due to the weak spin-orbit coupling and long spin scattering length [6]. The carbon atoms on two sublattices are spin-polarized as two-dimensional (2D) graphene contacts with ferromagnetic metal (FM) [7–9]. Spin-polarized transport behaviors were predicted theoretically in a 2D graphene-FM interface, for both in-plane [10–13] and out-of-plane electron transport [14, 15]. On the other hand, FM-graphene-FM-based spin valve devices were also fabricated experimentally to measure the spin-polarized transport in the direction perpendicular to the graphene plane [16–19]. More recently, both theoretical [20] and experimental studies [21] have found efficient spin injection for the FM-graphene system with the insertion of *h*-BN layers, though the spin-polarized current is greatly reduced due to the high tunneling barrier of *h*-BN [20]. However, 2D graphene has conical points located at the Fermi energy (*E*_F_) with zero density of states; the gapless property limits its application in half-metallic materials.

It is well known that the energy gap can be engineered by cutting a 2D graphene sheet into a one-dimensional (1D) graphene nanoribbon (GNR), where the edge carbon atoms are passivated by hydrogen. Recently, this nanometer-wide GNR with atomically precise width can be achieved via a surface-assisted bottom-up fabrication [22, 23]. Owing to the quantum confinement, electronic states of GNR are mainly governed by the boundary conditions [24, 25]. Consequently, the function of such a graphene nanoribbon-based device is strongly dependent on the edge structures.

One typical structure of the nanoribbon is the zigzag edge, referred to as a zigzag graphene nanoribbon (ZGNR), which shows flat-band magnetism induced by peculiar localized electronic states at each edge. Recently, the first-principles calculations predicted that anti-ferromagnetic ZGNR shows half-metallicity at a finite external electric field across the ribbon [3, 4]. Motivated by this approach, some alternative methods are also proposed to drive ZGNRs into the half-metallic state, such as edge modification by organic molecules [26], doping B/N atoms [27, 28], and adsorption ferroelectric polymer [29], acceptor/donor functional groups [30], or symmetric- and asymmetric-edge hydrogenations [31]. All these studies indicate that ZGNRs hold promising applications in the spintronic nanodevice.

The other signature shape is the armchair edge, termed as an armchair graphene nanoribbon (AGNR). Although AGNR has a band gap at the Fermi energy depending on the ribbon width, the electronic structure of AGNR is not spin-polarized [32]. Therefore, it is hard to fabricate spintronic devices based on the free-standing AGNR without other modification to control the spin of electrons. On the other hand, when AGNR is adsorbed on metals, such as Au, Cu, and so on, the electronic structure is modified by the interfacial coupling between the metal surface and graphene [23, 33]. Especially contacting with ferromagnetic metal onto the AGNR layer seems to be a feasible way of introducing spin polarization of the AGNR. However, the possibility of the AGNR system as spintronics has not been fully investigated.

**Fig. 1:**
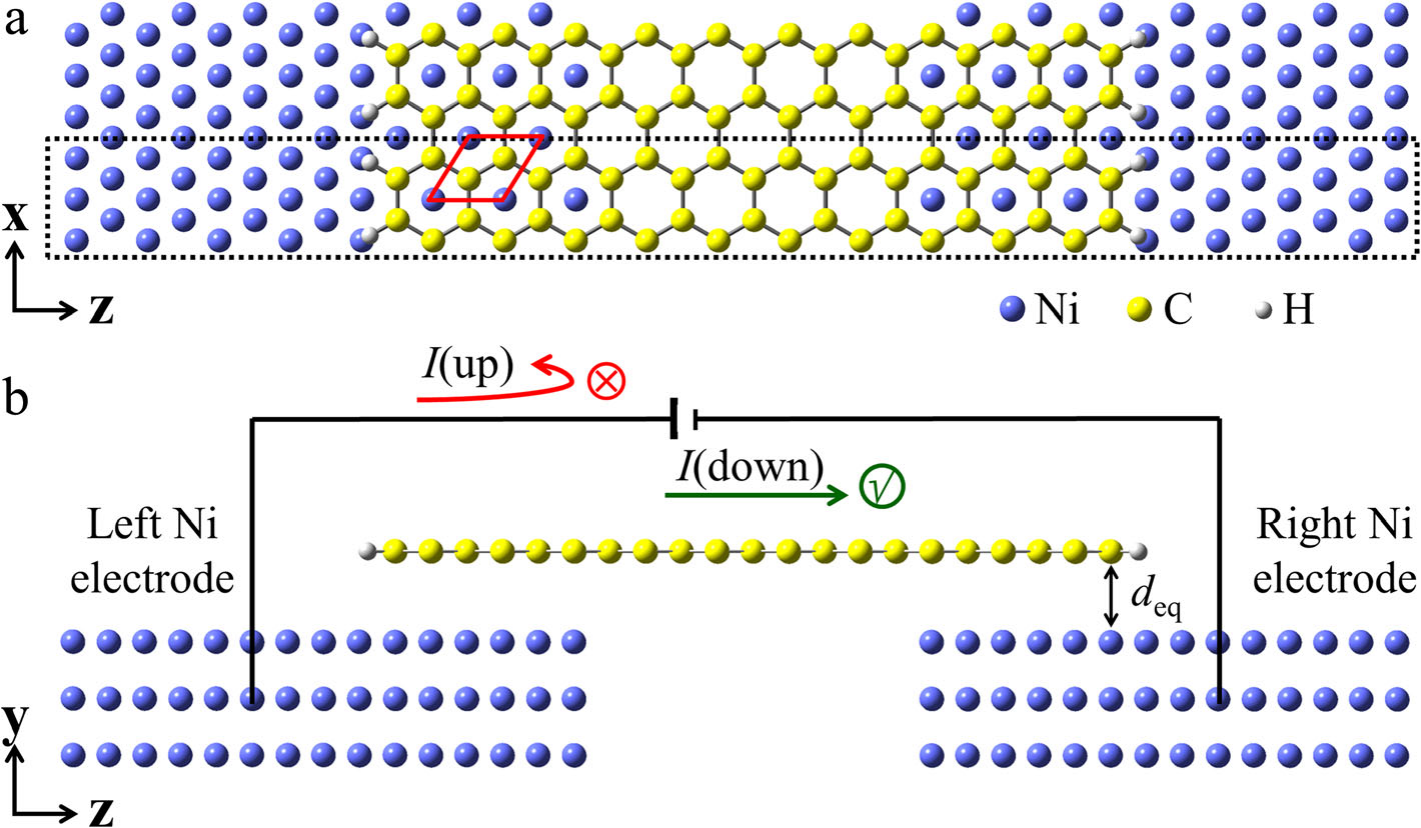
Scheme of the Ni/AGNR/Ni junction. **a** Top view of the *x-z* plane and **b** side view of the *y-z* plane. The armchair graphene nanoribbon with terminal edges passivated by hydrogen is adsorbed on Ni(111) surfaces. The junction is labeled as M3, which denotes three units of overlap at each edge and four units in the bridge part. The *blue*, *yellow*, and *white* balls denote Ni, C, and H atoms, respectively. The *dotted rectangle* shows the unit cell used in transport calculation, and the *red diamond* denotes the unit cell used in structure optimization. *d*_eq_ is the optimized interlayer distance between the AGNR and the Ni(111) surface

In this paper, we demonstrated that the half-metallic property can be realized in the non-magnetic AGNR via contact with Ni substrates (Ni/AGNR/Ni junction). The electron transport property of a two-probe junction, where AGNR is adsorbed on Ni(111) surfaces with an on-top configuration as shown in Fig. 1, was investigated by using first-principles theory. We found that half-metallicity in the Ni/AGNR/Ni junction depends on the interfacial coupling strength, such as interlayer distance and contact area.

## Methods

Figure 1 illustrates the studied model of a Ni/AGNR/Ni junction, constructed from three layers of Ni atoms with AGNR adsorbed on the surface. The unit cell used to model the junction is shown by the dotted rectangle in Fig. 1a. In the contact region, one carbon atom locates above a surface Ni atom and the other carbon is above a third-layer Ni atom. The contact area contains three repeating units at each side of the AGNR, and the junction is labeled as M3. The geometric structure of the contact region was firstly optimized with a 1 × 1 unit cell of graphene on three layers of Ni (111) surface (red diamond in Fig. 1a) using the density functional theory (DFT) code PHASE [34]. We used the PW91 functional parametrized by Perdew and Wang for the exchange-correlation term [35] and the Troullier-Martins-type atomic pseudopotentials [36, 37]. A plane-wave basis set with cutoff energy of 25 Ry was employed. A *k*-mesh of 20 × 20 × 1 was adopted to sample the Brillouin zone (BZ) for structural relaxations. During structural relaxation, the atoms are relaxed until the total energy change is less than 10^−9^ hartree and the force on each atom is smaller than 10^−3^ hartree/bohr.

In this structure optimization, we fixed the lattice constant of graphene to be the optimized value, *a* = 2.458 Å, adopting the same value for lattice constant of the Ni electrodes [38, 39]. With this orientation, the lattice mismatch between graphene and Ni(111) surfaces is about 1.3 %. Both C atoms and Ni atoms (except the Ni atoms in bottom layer) were relaxed to release the strain induced by lattice mismatch. The interlayer distance between the graphene and the Ni(111) surface is optimized to be 1.995 Å, as noted by *d*_eq_ in Fig. 1(b). This predicted distance of Ni-graphene is close to the experimental value (2.11 ± 0.07 Å) [40], previous LDA reports (2.05 Å) [7, 41] and the DFT calculation with van der Waals (vdW) correction (2.07 Å) [42], in which vdW density functional together with the C09 exchange functional was used.

The spintronic transport calculations were carried out by using the ASCOT code [43, 44] with a non-equilibrium Green’s function (NEGF) method [45] based on the DFT. In the present calculation, the magnetic moments of both ferromagnetic electrodes were aligned parallel to each other. Electron transport occurs in the *z* direction, and periodic boundary conditions are imposed in the transverse transport direction (*x* direction). The vacuum region in the *y* direction is ~10 Å to ensure decoupling between neighboring slabs. We employed the same functional and atomic pseudopotentials as the structure optimization procedure. For a basis set, we employed pseudoatomic orbitals [46], whose cutoff radius and number of primitive orbitals are summarized in Table 1. The energy cutoff for the real space mesh is 100 Ry for the transport calculation. For the self-consistent calculation of the two-probe open system, a *k*-point mesh of 19 × 1 × 1 was employed along the *x*, *y*, and *z* directions. The NEGF-DFT self-consistency is achieved when the energy difference is less than 10^−6^ hartree between the iteration steps. For the calculation of the current and the spin-decomposed transmission spectra, that is defined as

1 $$ {T}_{\sigma }(E)=\frac{1}{N_k}{\displaystyle \sum_k{T}_{\sigma}\left(k,\kern0.5em E\right)} $$

**Table 1:** Cutoff radius *r*_c_ of the pseudoatomic orbitals and number of primitive orbitals for *s*-, *p*-, and *d*-orbitals (*n*_s_, *n*_p_, *n*_d_)

	*r*_c_ (a.u.)	*n*_*s*_	*n*_*p*_	*n*_*d*_
Ni	6.0	2	1	1
C	6.0	2	2	–
H	4.0	2	2	–

We used *k*-point *N*_k_ = 201 in the periodic *x* direction, and 1 in the *y* and *z* directions. The energy cutoff and *k*-point convergences were performed for all calculations.

## Results and Discussion

**Fig. 2:**
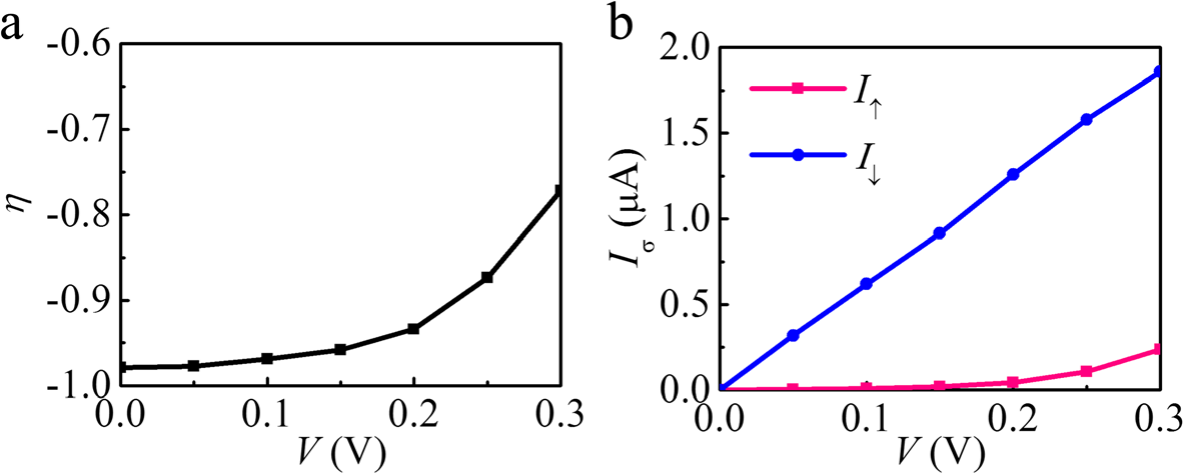
**a** The spin injection factor *η* against bias *V* for M3. **B** The spin-decomposed current-voltage (*I*_σ_-*V*) curves of M3

To show the spintronic transport properties of Ni-contacted AGNR, Fig. 2a, b display the spin filter characteristics of the M3 and the spin-decomposed current-voltage curves, respectively. The spin injection factor *η* is defined as *η* = (*I*_↑_ − *I*_↓_)/(*I*_↑_ + *I*_↓_), where *I*_σ_ is the current of each spin σ(=↑,↓). In the bias voltage range *V* < 0.3 V, the absolute value of *η* is more than 0.77. Remarkably, a high spin-filtering effect, *η* of −0.98, is observed at a low bias voltage. This indicates that the current flow in the Ni-contacted AGNR is highly spin-polarized and, as shown in Fig. 2b, the total current is dominated by down spin due to the rather low *I*_↑_, showing a metallic behavior for the down spin while a nearly insulating behavior for the up spin. Notably, the nearly 100 % spin-filtering efficiency is obtained in the junction composed of AGNR without intrinsic magnetism, suggesting that half-metallicity can be realized in a non-magnetic molecule via contact with ferromagnetic metal electrodes.

**Fig. 3:**
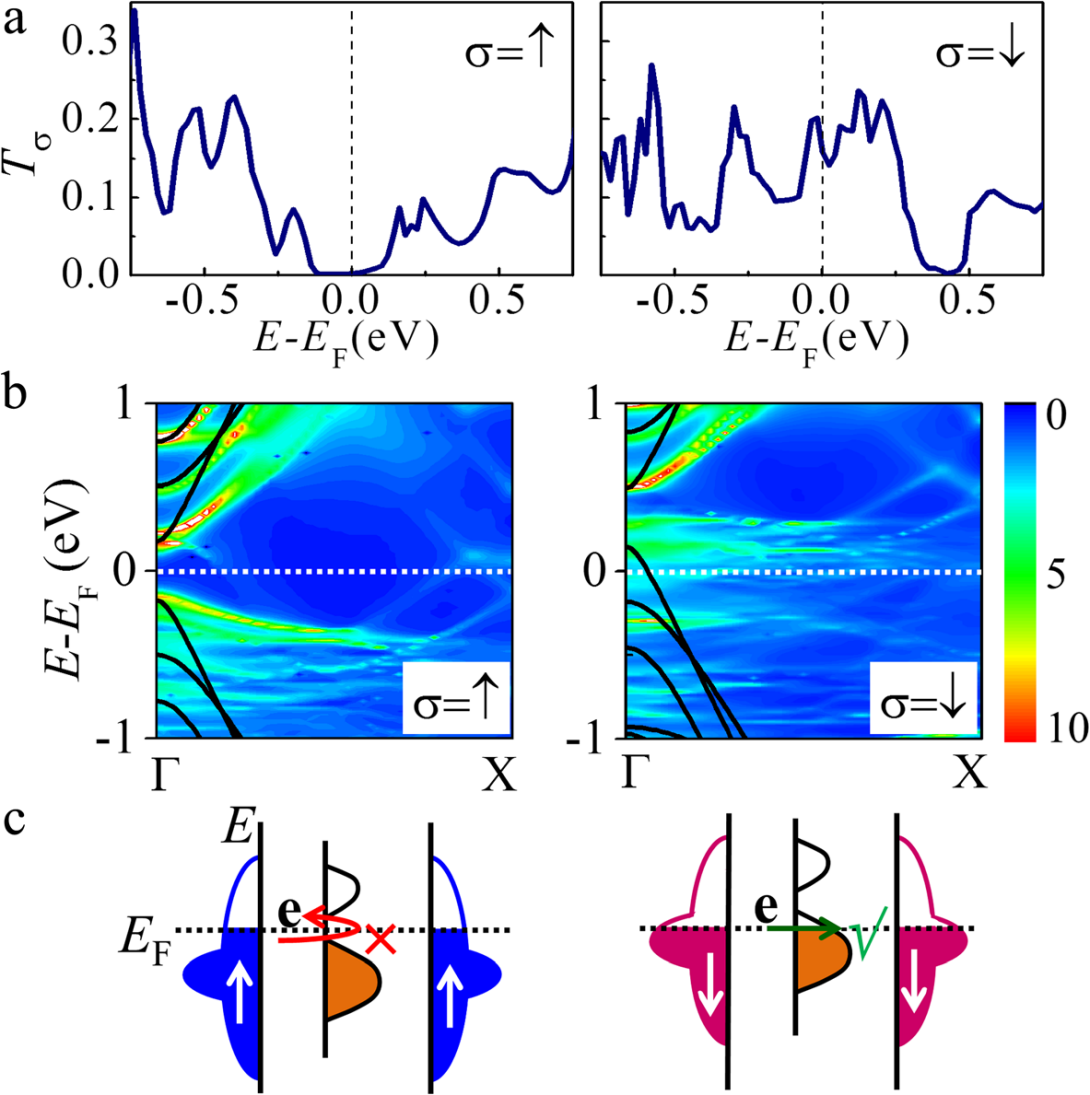
**a** Spin-dependent transmission spectra for M3 at zero bias. The Fermi energy is set to 0 eV shown by *vertical dotted lines*. **b** The LDOS with respect to the AGNR in the M3 junction in the BZ of a unit cell. For a clear comparison, the band structure of the isolated AGNR is shown together by *solid lines*. The conduction band bottom of the isolated AGNR is set to be the projected LUMO energy level at the Γ point. **c** Schematic view of the state alignment of AGNR for M3. A gap is opened at *E*_F_ for up spin due to the energy gap of AGNR, while electronic states are generated at *E*_F_ for down spin as the strong hybridization. Results of up (down) spin are shown in *left* (*right*) *panels*

In order to clarify the origin of the half-metallic property, we show the spin-dependent transmission spectra of the M3 junction in Fig. 3a. At *E*_F_, it is found that for up spin, there is a zero-transmission gap with a width of ~0.2 eV, while for down spin, a high transmission coefficient of 0.16 is observed and the zero-transmission gap locates at ~0.4 eV. From the local density of states (LDOS) of AGNR in the Brillouin zone (BZ) and the band structure of an isolated AGNR as shown in Fig. 3b, it can be clearly seen that AGNR states are spin-polarized due to the interaction with Ni substrates. In the middle *k*-region of the BZ, high states exist in the LDOS, which are induced by strong hybridization between Ni *d*-states and graphene π-orbitals [38, 39]. At *E*_F_, in particular, these generated states are more obvious for down spin, since Ni *d*-states of the down spin locate in a higher energy region than that of the up spin. This is the reason for the higher transmission observed for the down spin. In addition, by comparing transmission spectra and LDOS, we find that the transmission gap arises from the band gap of the isolated AGNR. The location of the gap closely relates to the interfacial charge transfer intensity, which depends on the strength of the Ni-AGNR interaction, as reported for the Ni-AGNR system in the previous work [39]. In the M3 system, electrons of 1.43 e transfer from AGNR to Ni substrates and the predicted charge transfer direction is in agreement with the experimental result [47]. These features are schematically described in Fig. 3c. In this way, around *E*_F_, the transmission of up spin becomes almost zero due to the AGNR gap located at *E*_F_, while a higher transmission of down spin is observed because of strong hybridization.

As mentioned above, to realize this half-metallicity, it is necessary to drive the gap of the up spin to reside at *E*_F_. The gap location is determined by the strength of interfacial interaction which directly correlates with, for example, the interlayer distance between AGNR and the metal surface and the contact area, because the interaction strength can alter the amount of charge transfer which relates to the gap location. This indicates that the efficiency of the spin filter might be strongly dependent on the interaction strength.

**Fig. 4:**
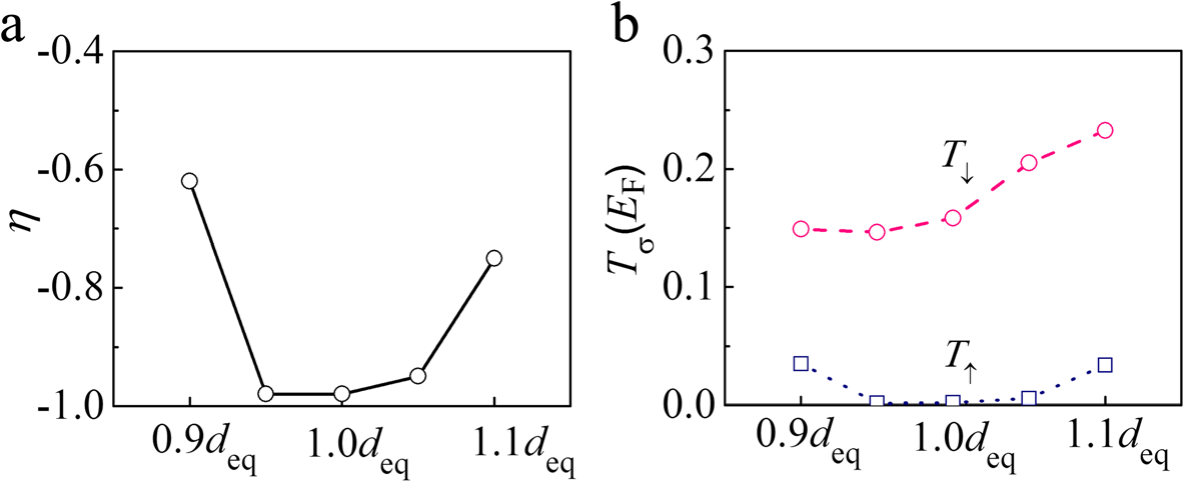
**a** Spin injection factor *η* and **b** transmission coefficient at *E*_F_ [*T*_σ_(*E*_F_)] of M3 with various interlayer distances at zero bias

In order to confirm these effects, first, we explore the spin-filtering effect of the M3 junction with different distances varying from 0.90*d*_eq_ to 1.10*d*_eq_, where *d*_eq_ is the equilibrium distance between AGNR and the Ni surface (1.955 Å). Figure 4a, b displays the *η* values and the transmission coefficient at *E*_F_ [*T*_σ_(*E*_F_)] of all contact structures at zero bias, respectively, where *η* at zero bias is defined as *η* = [*T*_↑_(*E*_F_) − *T*_↓_(*E*_F_)]/[*T*_↑_(*E*_F_) + *T*_↓_(*E*_F_)]. The spin injection factor of M3 retains −0.98 as the distance is shortened to 0.95*d*_eq_, and then *η* drops down for a much smaller spacing of 0.90*d*_eq_. For a larger distance, *η* is reduced as the distance extends to 1.10*d*_eq_. This variation of *η* is mainly attributed to the increase of the up-spin transmission coefficient at *E*_F_ for a shorter or larger distance. As shown in Fig. 4b, *T*_↑_(*E*_F_) increases from 0.002 (1.00*d*_eq_) to 0.035 (0.90*d*_eq_) or 0.034 (1.10*d*_eq_). The evolution of *η* and the transmission coefficient can be understood from the location of the AGNR band gap in energy.

**Fig. 5:**
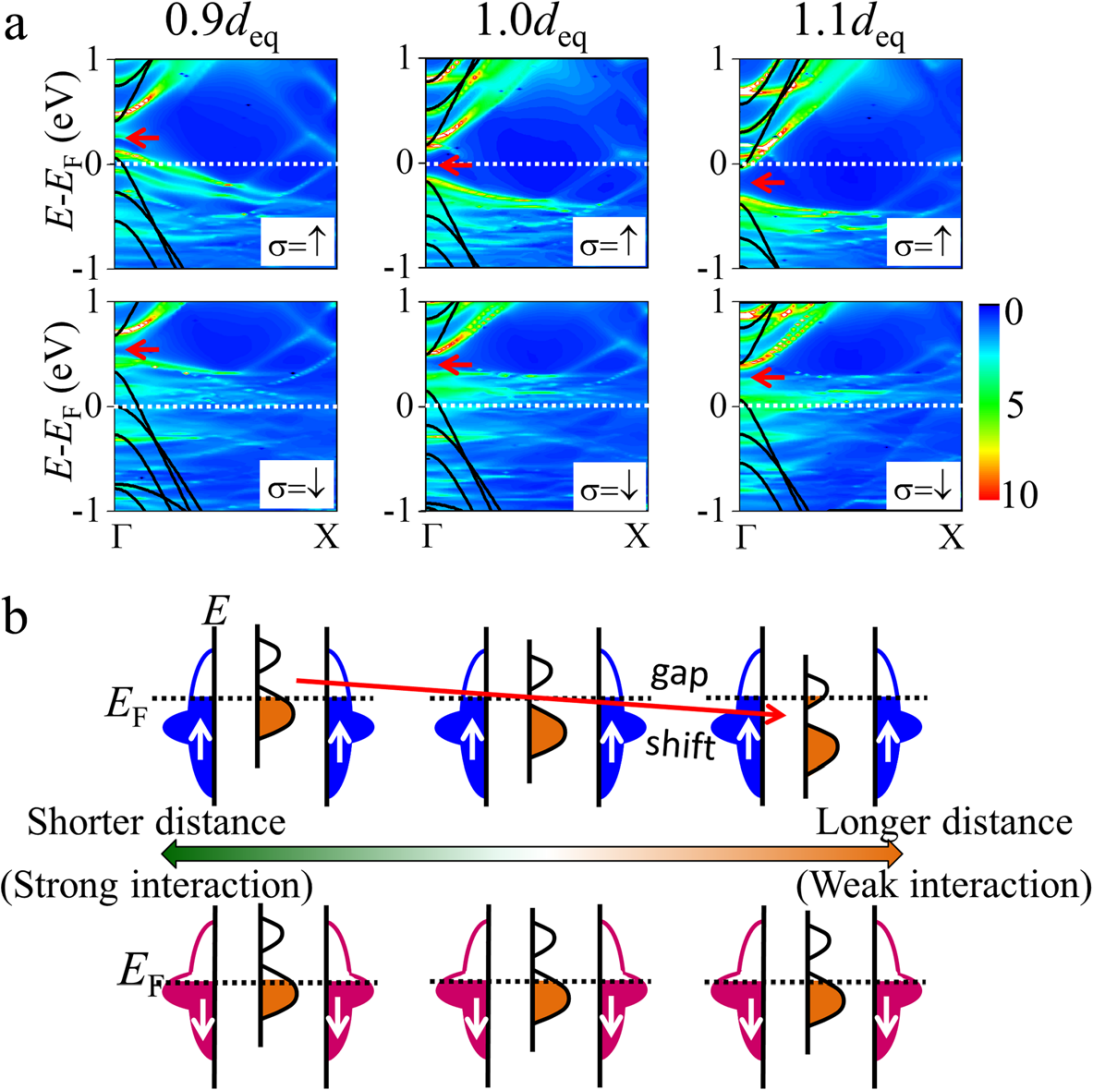
**a** The LDOS with respect to the AGNR in junctions and the band structure of an isolated AGNR. The conduction band bottom of the isolated AGNR is set to be the projected LUMO energy level at the Γ point. The *arrows* denote the location of the energy gap. **b** Schematic view of the state alignment of AGNR. From *left* to *right*, models of 0.90*d*_eq_, 1.00*d*_eq_, and 1.10*d*_eq_ are shown. Schemes of up (down) spin are shown in the *upper* (*lower*) *panel*. For up spin, the shift of gap location as the distance changes is denoted by the *arrow*

To gain insight into the gap location, we show the *k*-dependent LDOS and band structure of an isolated AGNR in Fig. 5a. As the distance becomes shorter than the equilibrium distance, the gap shifts to the upper energy side, from 0 eV (0.4 eV) of 1.00*d*_eq_ to 0.25 eV (0.55 eV) of 0.90*d*_eq_ for the up (down) spin as noted by the arrows. In contrast, weaker interaction for a longer distance causes an opposite shift of the band gap, and the gap approaches −0.2 eV (0.3 eV) of 1.10*d*_eq_ for the up (down) spin. Figure 5b schematically describes the gap shift, which relates to the variation of interfacial charge transfer intensity, indicating that a stronger interaction leads to a larger amount of charge transfer from AGNR to Ni electrodes. With increase or decrease of the spacing distance, the gap of the up spin gradually moves away from *E*_F_, raising the up-spin transmission value at *E*_F_, and as a result, the efficiency of the spin filter is reduced.

**Fig. 6:**
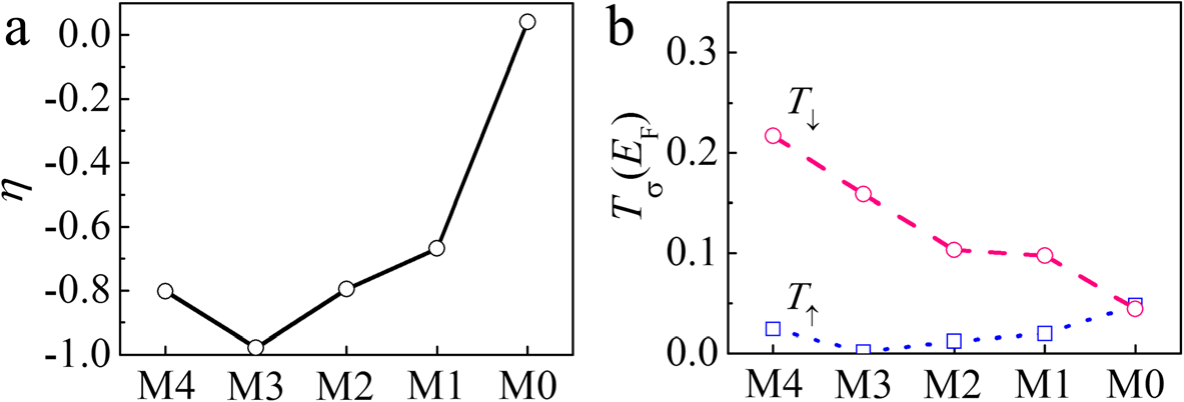
**a** Spin injection factor *η* and **b** transmission coefficient at *E*_F_ [*T*_σ_(*E*_F_)] of Ni/AGNR/Ni junctions with different contact areas at zero bias

Next, we study the spin-filtering effect of Ni/AGNR/Ni junctions with different contact areas. The contact area varies from no overlap (M0) to four repeating units of overlap at each side (M4). Figure 6a displays the *η* values of all contact structures. It is clearly seen that M3 exhibits the largest *η* value of −0.98 and the absolute value of *η* becomes smaller with reducing contact area, and then we obtain *η* of nearly zero for the M0 system. This contact area dependence arises from the following properties of *T*_σ_(*E*_F_) as shown in Fig. 6b. As the contact area decreases, *T*_↓_(*E*_F_) monotonically decreases from 0.16 for M3 to 0.044 for M0. In contrast, *T*_↑_(*E*_F_) shows an opposite trend which is slightly enhanced and a minimum value (0.002) is found for the up spin of the M3 system. For M0, the spin dependence is so weak that *T*_σ_(*E*_F_) is similar for two spin states.

**Fig. 7:**
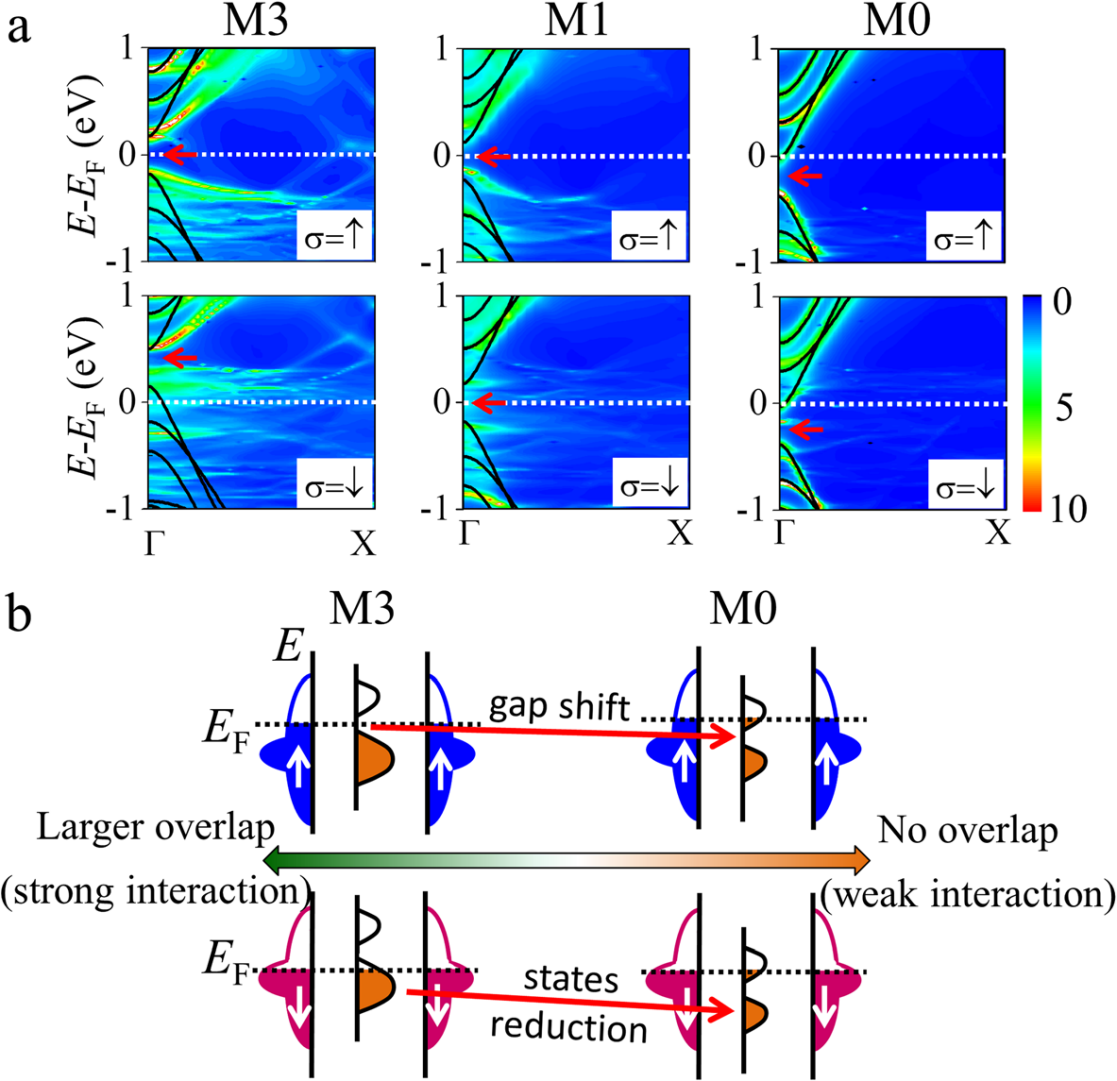
**a** The LDOS with respect to the AGNR in junctions (M3, M1, and M0) and the band structure of an isolated AGNR. The conduction band bottom of the isolated AGNR is set to be the projected LUMO energy level at the Γ point. The *arrows* denote the location of energy gap. **b** Schematic view of the state alignment of AGNR for M3 and M0. Schemes of up (down) spin are shown in the *upper* (*lower*) *panel*

Figure 7a shows the LDOS and band structure of the isolated AGNR in M3, M1, and M0 systems. With varying of the contact area from M3 to M0, the AGNR gap located at *E*_F_ for up spin shifts to the lower energy side with the reduction of charge transfer intensity. This downward shift of the AGNR gap gives rise to a slight increase of the up-spin transmission at *E*_F_, whereas weaker interaction reduces the electronic states of the down spin and induces a lower transmission at *E*_F_. The effects of the gap shift and state reduction, which are observed above, are schematically depicted in Fig. 7b. On the other hand, the spin-filtering effect of a case of more overlap, that is, M4, is weaker than that of M3. This is because the stronger interfacial interaction of M4 enhances the transmission of up spin, while the gap location in energy is almost same as M3. The above results suggest that the spin-filtering effect depends strongly on the size of the contact region, which can also change the AGNR gap location, same as the origin of the distance-dependent *η*. As a consequence, we can confirm that the efficiency of the spin filter is strongly dependent on the strength of the AGNR-Ni interaction. To achieve a high efficiency, a moderate interaction strength which makes the AGNR gap locate at *E*_F_ for only one spin is required.

At last, it is emphasized that the present system utilizes the non-magnetic carbon nanomaterial in contact with magnetic substrates, distinct from previously predicted half-metals with intrinsic magnetism, such as manganese perovskites [1], Heusler compounds [2], and recently discovered metal-free half-metals (for example, graphitic carbon nitride) [5]. These results may bring us the possibility of fabricating spintronic nanodevices based on non-magnetic 1D graphene nanoribbon by interfacial manipulation.

## Conclusions

In summary, we observe the half-metallic property in an armchair graphene nanoribbon in contact with Ni electrodes. The junction exhibits a spin injection value of −0.98, indicating a nearly insulating behavior for up spin and a metallic behavior for down spin. This spin-filtering effect originates from the following mechanisms: Owing to the interaction between AGNR and Ni states, the AGNR energy gap of up spin is located at *E*_F_, suppressing the up-spin transmission, while a large transmission of down spin is observed at *E*_F_. The efficiency of the spin filter varies with the AGNR gap location, which correlates with the charge transfer intensity determined by the interaction strength between graphene and the Ni surface, that is, the interlayer distance and contact area. This device design suggests a potential application of AGNR-based materials in spintronic nanodevices.
